# Generating a heterosexual bipartite network embedded in social network

**DOI:** 10.1007/s41109-020-00348-1

**Published:** 2021-04-12

**Authors:** Asma Azizi, Zhuolin Qu, Bryan Lewis, James Mac Hyman

**Affiliations:** 1 Department of Mathematics, University of California, Irvine, CA 92697 USA; 2 Department of Mathematics, The University of Texas at San Antonio, San Antonio, TX 78249 USA; 3 Biocomplexity Institute and Initiative, University of Virginia, Charlottesville, VA 22904 USA; 4 Department of Mathematics, Tulane University, New Orleans, LA 70118 USA

**Keywords:** ***B*2*K*** network, Bipartite network, Social contact network, Heterosexual network, Joint degree distribution, Sexually transmitted infections

## Abstract

We describe an approach to generate a heterosexual network with a prescribed joint-degree distribution embedded in a prescribed large-scale social contact network. The structure of a sexual network plays an important role in how all sexually transmitted infections (STIs) spread. Generating an ensemble of networks that mimics the real-world is crucial to evaluating robust mitigation strategies for controlling STIs. Most of the current algorithms to generate sexual networks only use sexual activity data, such as the number of partners per month, to generate the sexual network. Real-world sexual networks also depend on biased mixing based on age, location, and social and work activities. We describe an approach to use a broad range of social activity data to generate possible heterosexual networks. We start with a large-scale simulation of thousands of people in a city as they go through their daily activities, including work, school, shopping, and activities at home. We extract a social network from these activities where the nodes are the people, and the edges indicate a social interaction, such as working in the same location. This social network captures the correlations between people of different ages, living in different locations, their economic status, and other demographic factors. We use the social contact network to define a bipartite heterosexual network that is embedded within an extended social network. The resulting sexual network captures the biased mixing inherent in the social network, and models based on this pairing of networks can be used to investigate novel intervention strategies based on the social contacts among infected people. We illustrate the approach in a model for the spread of chlamydia in the heterosexual network representing the young sexually active community in New Orleans.

## Introduction

The structure of heterosexual networks plays an important role in the spread of all sexually transmitted infections (STIs), including chlamydia and gonorrhea. These networks are captured in computer simulations by a bipartite graph where the nodes represent the people and the edges are sexual partnerships between nodes of different sexes. Determining what is predictable in STI models requires an algorithm to generate an ensemble of random graphs that resembles real-world sexual activities. These graphs must account for the distribution for the number of sexual partners people have (their degree distribution) and the number of partners their partners have (the joint-degree, or degree-degree, distribution). The existing algorithms that generate bipartite random graphs preserving degree and joint-degree distributions of the nodes are strictly based on the number of partners people have and not other demographic factors, such as age or location (Newman 2002; Hakimi 1962; Boroojeni et al. 2017; Azizi et al. 2016, 2017, 2018).

The degree and joint-degree distributions are just two of many properties for a heterosexual network that can affect its structure and the validity of an epidemic model. The heterosexual network is also correlated to an underlying *social contact network* of acquaintances connected by interpersonal relationships. A person’s sexual activity depends on age, race, sociodemographic, and socioeconomic features of the environment that can be captured by a social contact network (Amirkhanian 2014; Adimora and Schoenbach 2005; Ruan et al. 2011; Juher et al. 2017; Morris et al. 1995). In other words, using the extended social network of a person as a source of sexual partner selection when generating a heterosexual network enables the network to capture the bias in heterogeneous mixing based on age, race, economic status, and geographic location (McPherson et al. 2001).

Although it is widely accepted that social contact (non-sexual partners) and heterosexual (sexual partners) networks are related, there are few studies on how a population’s social contact network impacts the spread of heterosexual STIs. The social network can affect the structure of the heterosexual network by providing a pool of sexual partners. A persons social network also influences their cultural norms regarding STI testing, safe sex practices, and knowledge about the spread and treatment of STIs. As far as we know, there are no other existing mechanistic approaches that construct a heterosexual network embedded within a realistic social network. We will describe a new approach that fills this gap by applying social contact networks to generate the heterosexual network while preserving the joint-degree distribution of data.

Our new network generation approach uses the underlying extended social network of a population to extend these previous algorithms for generating bipartite heterosexual networks with prescribed joint-degree distribution (Boroojeni et al. 2017). Many sexual partnerships are formed from within a person’s social circle, defined by the people they have regular social contact with and the contacts of their contacts (their extended social network). These social circles have been modeled through large-scale simulations of thousands of people in a city as they go through their daily activities. We start with a network that mimics the social activity of the population (Eubank et al. 2010), as generated by a complex social network simulation. We use this simulated data to create an extended social network and then identify a bipartite network of men and women to define our heterosexual network. We then create a virtual heterosexual network as a subgraph of this bipartite social network that captures a prescribed joint-degree distribution.

As a case study, we construct a heterosexual network that is embedded in the social contact network of the New Orleans population and mimics the sexual behavior obtained from a sexual behavior survey of the young adult African American population in New Orleans (Kissinger 2014; Green et al. 2014).

## Materials and method

People often find their sexual partners within their extended social network, the individuals they contact each day at work, school, or other social activities. There are sophisticated simulations of these social networks , such as EpiSims that is based on the Transportation Analysis and Simulation System (TRANSIMS) developed at Los Alamos National Laboratory (Barrett et al. 2005) or *Simfrastructure* (Eubank et al. 2010; Eubank 2008). These social networks can be used to produce a sexual network, which is more realistic than basing partnerships on just the sexual activity of different individuals.

The social contact network is a graph where the nodes are synthetic people, labeled by their demographics (sex, age, income, location, etc.), and the edges between the nodes represent contacts determined in which each synthetic person is deemed to have made contact with a subset of other synthetic people through some *Activity* types. Each edge of the network is labeled with one of these activity locations and is weighted by the time spent on these contacts per day. For example edge (**i**,**j**) labeled by the activity $A=Work$ and weighted by $T^W_{i,j}$ means two persons **i** and **j** have a contact for $T^W_{i,j}$ fraction of their total time spent at work. We base our algorithm on a social contact network, called **SocNet**, generated by Eubank et al. (2010) with activity at different locations (e.g., home, work, school, shopping, or other activity).

We introduce an algorithm that embeds a heterosexual network within a social network and matches the sexually active population’s joint-degree distribution. The heterosexual network preserves the bipartite joint degree (*BJD*) distribution matrix that represents the correlations between then number of partners a person has and the number of partners their partners have (Boroojeni et al. 2017). The algorithm has three stages:

(i) Generate an extended social contact network, **ESocNet**: The original social contact network is a simple graph, whose nodes are synthetic people, and neighboring nodes are their social contacts during a typical day. We assume that most sexual partnerships come from a person’s social contacts or the social contacts of their social contacts, e.g., the neighbors of the neighbors of a node. We extend the social contact network to create a new network, the extended social network, **ESocNet**, where some of the neighbors (social contacts) of an individual’s neighbors in this network are added to his/her social contacts.
(ii) Generate a reduced social bipartite network, **BSocNet**: The **ESocNet**  includes all the individuals in the region being modeled. Our sexual network is based on individuals within a prescribed age range. In this step, we remove all nodes where the associated individuals are outside this age range. The extended social network is a simple graph where nodes have some neighbors that are of the same sex. We identify the embedded bipartite subgraph of this network by removing all edges between individuals of the same sex. The resulting bipartite graph is a social network where male nodes are only connected to female nodes and vice versa.
  Finally, we removed monogamous couples from the simulation since they are not part of the STI transmission network and assume that siblings are not sexual partners. This was achieved by removing all edges between individuals living in the same household, which is the edge labeled activity H for home. This is an approximation since there are some non-monogamous couples living in the same household, even in the high-risk young adult African American population being modeled. We call this reduced social bipartite network as **BSocNet**.
(iii) Generate an embedded heterosexual bipartite network, **SexNet**: We then use the **BSocNet**  to define a heterosexual network of sexual partnerships, the **SexNet**, with a prescribed bipartite joint degree matrix *BJD* based on survey data (Boroojeni et al. 2017). That is, we preserve the correlations between the number of partners a person has and the distribution for the number of partners their partners have. We assume that most of a person’s sexual partners are neighbors in the **BSocNet**  and a few of the partners are randomly selected from elsewhere in the population where they might have met through social media or at any other event.

### Generate an extended social contact network (**ESocNet**)

In the first stage of our algorithm, we create an extended social contact network, **ESocNet**, so that an individual’s social contacts include some of the contacts of their contacts. That is, **ESocNet**  will add potential sexual partners by including some of the social contacts of an individual’s social contacts.

Consider two people (nodes) **i** and **j** who are not currently connected, but have $k_A(i,j)>0$ common social contacts within activity *A*. We define $p^A_{ij}$ as the probability that they will meet through a single contact. Therefore, the probability that they will meet and be connected in the **ESocNet**  after $k_A(i,j)$ contacts is $1-(1-p^A_{ij})^{k_A(i,j)}$.

**Fig. 1:**
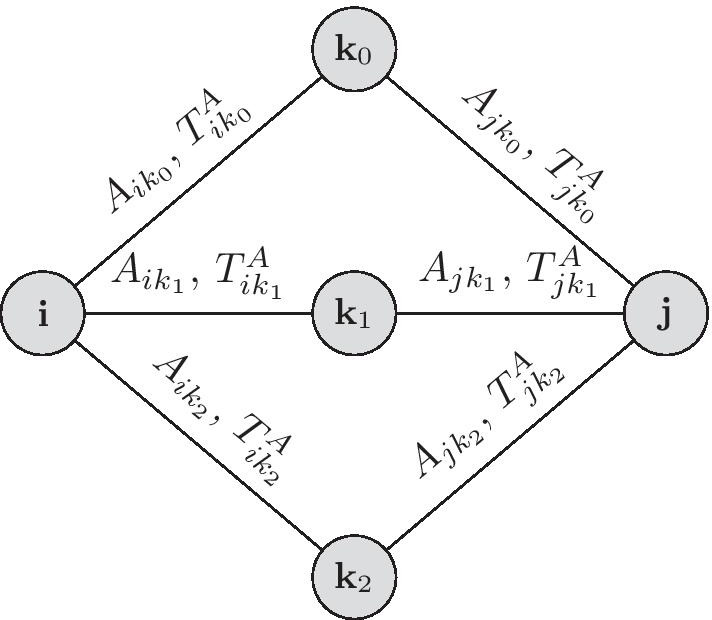
Suppose the persons **i** and **j** are not currently social contacts in **SocNet**but have three different common social contacts **k**_0_,**k**_1_, and **k**_2_ through different activities. They might be connected in the extended social network, **ESocNet**, when at least one of their common social contacts meet them within the same activity location. Suppose $A_{ik_0}=A_{jk_0}=A_{ik_1}=A_{jk_1}=A\ne A_{ik_2}\ne A_{ik_2}$, that is, **k**_0_ meets **i** and **j** at the same location, similarly **k**_1_ meets **i** and **j** at the same location to **k**_0_’s, however, **k**_2_ meets them in different places. To compute $p^A_i$ and $p^A_j$ we only count the social contacts who meet them at the same location A, therefore, $p^A_i=(T^A_{ik_0}+T^A_{ik_1})/{2}$, and $p^A_j=(T^A_{jk_0}+T^A_{jk_1})/{2}$. Finally, the probability that **i** and **j** make an edge- are connected in the extended social network- is $1-(1-p^A_ip^A_j)^{2}$

The probability $p^A_{ij}$ is a function of the time that **i** and **j** spend in an activity *A* in **SocNet**. From the data in **SocNet**, we can define $\tau ^A_k$ as the average fraction of time person **k** spends with each social contact, when engaged in activity *A*:

1 $$\begin{aligned} \tau ^A_k=\frac{\sum _{l \in N_A(k) } T^A_{k,l}}{|N_A(k)|}~, \end{aligned}$$

where $T^A_{k,l}$ is the fraction of time two contacts **k** and **l** spend together in activity *A*, and $N_A(k)$ is set of all social contacts for person **k** through an activity location *A*. We then define $p^A_{ij}=\tau ^A_i \tau ^A_j$. Figure 1 describes a schematic of this algorithm for a simple network.

### Generate a reduced social bipartite network, **BSocNet**

In our heterosexual network, we only consider the sexually active population within a prescribed age range $\alpha =[\alpha _1,\alpha _2]$. That is, we trim the **ESocNet**  by removing all people with ages outside this range to not including any edges (sexual contacts) with people outside this range. We then remove all edges between people of the same sex to create a bipartite heterosexual social network. To avoid including siblings as potential sexual partners and because men and women in our young high-risk population cohort are less likely to live together as sexual partners in the same household, we eliminate the sexual partnerships between individuals living in the same household by removing all edges labeled activity H as home, to define the **BSocNet**.

### Generate an embedded heterosexual network (**SexNet**)

The $\mathbf{Soc2Sex}$ algorithm uses **BSocNet**  to generates a heterosexual network, **SexNet**, that mimics the heterogeneous mixing of the real population. We assume that we have an estimate for the distribution for the number of partners of men and women (the degree distributions for their associated nodes) and the joint-degree distribution for the number of partners that their partners have (Boroojeni et al. 2017).

An edge, $\mathbf{ij}$, between two persons **i** and **j** in **SexNet**  represents a sexual partnership. The *degree* of a person **i**, is defined by the number of his/her sexual partners. The *degree distribution* $\{d_k\}$ defines the number of people with degree *k*. The *joint-degree distribution* (*k*, *j*) is the number of partnerships between a man with degree *j* and a woman with degree *k*. This distribution can be represented by the *Bipartite Joint Degree* or *BJD* matrix:

$$\begin{aligned} BJD_{\mathbf{SexNet}}=\begin{pmatrix} e_{11} &{} e_{12} &{} e_{13} &{} \cdots &{} e_{1m}\\ e_{21} &{} e_{22} &{} e_{23} &{} \cdots &{} e_{2m}\\ &{}&{}{\vdots } &{}&{} \\ e_{w1} &{} e_{w2} &{} e_{w3} &{} \dots &{} e_{wm} \end{pmatrix}, \end{aligned}$$

where, *w* is the maximum degree in women nodes, and *m* is the maximum degree in men nodes, each element $e_{ij}$ is the number of edges between women with *i* partners and men with *j* partners.

The degree distribution of the number of women nodes, $d^w_k$, and men nodes, $d^m_k$, with *k* partners can be obtained from $BJD_{\mathbf{SexNet}}$:

2 $$\begin{aligned} d^w_k=\frac{\sum _{j=1}^m e_{kj}}{k}~,\text { and }~~~d^m_k=\frac{\sum _{i=1}^w e_{ik}}{k}~. \end{aligned}$$

Though the heterosexual network **SexNet**  is a subgraph of **BSocNet**, we also consider some sexual partners that are within a person’s extended social circle. That is, for the general case, **SexNet**  is partially embedded in **BSocNet**.

The $\mathbf{Soc2Sex}$ algorithm first generates an initial heterosexual network that closely agrees with the desired *BJD* matrix and is partially embedded in **BSocNet**. Usually, this network satisfies the desired *BJD*, but can fail when the average degree (number of partners people have) becomes large. When this happens, a second fix-up algorithm, based on rewiring the network, is used to repair any discrepancies so tht the final **SexNet**  has the desired *BJD* matrix.

#### Generating the bipartite network

The $\mathbf{Soc2Sex}$ algorithm starts with the **SocNet**, the *BJD* matrix corresponding to **SexNet**, and the fraction $p\in [0,1]$ of partners that are chosen randomly from the extended social contacts in the **BSocNet**. The remaining fraction, $(1-p)$, of a person’s sexual partners are randomly selected from elsewhere in the population. These partnerships might have formed by meeting through social media or a social event not captured by the original **SocNet**. The $\mathbf{Soc2Sex}$ algorithm then generates a heterosexual network that is a partial subgraph of **BSocNet**  and has a joint-degree distribution given by the *BJD* matrix. Note that *p* is approximately the percentage of **SexNet**  that is a subgraph of **BSocNet**.

The algorithm starts with an empty set of nodes **SexNet** and then builds a network guided by the *BJD* matrix. The nodes with the smallest degree have the least flexibility, so we start building **SexNet**  by randomly selecting a man node of **BSocNet**  with the highest (social) degree and assign its desired sexual degree to be column size of *BJD* matrix. This is represented by *stubs*, or unconnected edges, associated with this node.

We repeat the following process until all edges in **SexNet**, which is equal to the summation of elements in *BJD* matrix, are placed: at any step, we select a node with the highest stub in **SexNet**. Then we generate a uniform random number. If this random number was less than *p* then we use the pool of the node’s social contact in **BSocNet**find him/her a partner with proper degree defined by *BJD*. If the random number was bigger than *p*, we find the partner with proper degree defined by *BJD* from people other than their social contact but with smallest distance in their social contact. After finding such partner, we reduce the node’s new partner’s stub by one. If we find all the partners for all nodes in **SexNet**, we add a new node to **SexNet**  from highly socially active nodes in **BSocNet**. Then, we assign this new added node a desired degree and stub equal to the current maximum degree frequency of **SexNet**.

To keep or remove an edge, we have to calculate the degree of nodes attached to it for each possible edge in the **SocNet**, thus, the full set of experiments run in O($|E|P^mP^w$) time, where |*E*| is the number of edges in **SocNet**, $P^m$ number of its men nodes and $P^w$ number of its women nodes. This method is feasible if the average degree ($\frac{2|E|}{P^m+P^w}$) of the network is not high.

Table 1 shows the average CPU time for generating a **SexNet** with between 400 nodes to 12,800 nodes that are embedded within the **BSocNet**. These networks all maintained the same average degree, 1.5, and men-women ratio, 0.35:0.65, as our survey data.

**Table 1:** Average CPU time in seconds and 95% confidence intervals (CI) for generating 50 random **SexNet** on a MacBook Pro laptop computer. Notice that the CPU time scales almost linearly with the network size for a fixed average degree

Network size =	400	800	1600	3200	6400	12,800
CPU time	12.0	23.6	43.2	86.7	225	497
95% CI	[11.5, 12.4]	[23.3, 23.8]	[42.3, 44.2]	[86, 88]	[219, 230]	[454, 540]

For completeness, we provide pseudo-code for our Python scripts in Algorithms 1 and 2. Table 2 is the table of symbols used in these Algorithms.

**Table 2:** Table of notation for a conventional network *G* in algorithms

Notation	Description
*G*.*n*	Set of nodes in network *G*
*G*.*e*	Set of edges in network *G*
$d_G$	Degree frequency list for network *G*
$d_G({\mathbf{i}})$	Degree of node **i** in network *G*
$G.N({\mathbf{i}})$	Set of neighbors of node **i** in Network G
$dist(G,{\mathbf{u}},{\mathbf{v}})$	Distance between two nodes **u** and **v** in Network G
*M*.*col*/*M*.*row*	Column/row size of a matrix M
*M*(*i*, : )/*M*( : , *i*)	*i*th row/column of matrix M
*V*(*i*)	*i*th element of vector V
*V*.*index*(*a*)	Index of element a in vector V
|*S*|	Size (the number of elements) of a set S
*S*.*remove*(*m*)	Remove member m from a set S
*S*.*sample*(*P*)	Randomly select an element with property P (if P = 1 there is no property) from set S
*urn*	Uniform random number in [0, 1]


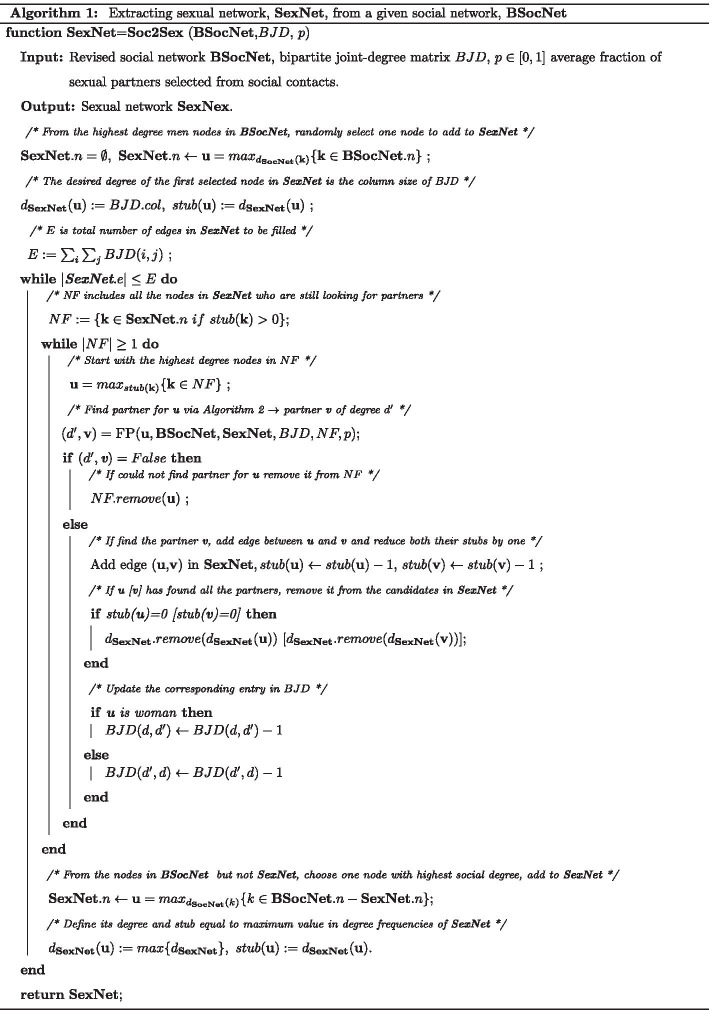

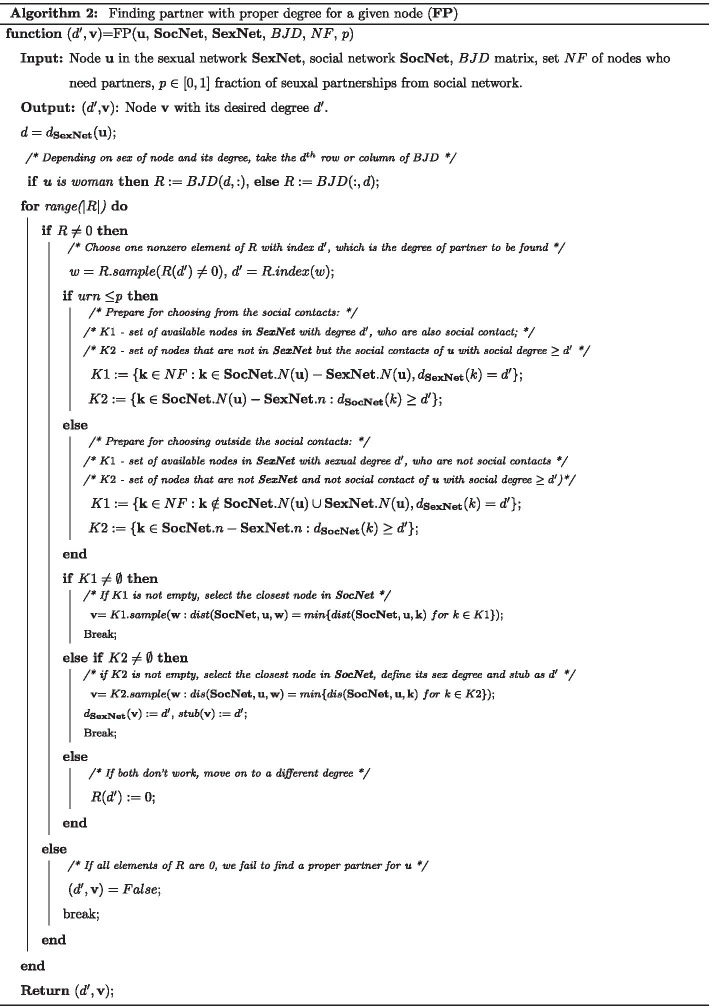


The initial algorithm will generate a network with the prescribed degree distribution. We have observed that the resulting network always had the desired *BJD* matrix for all of the sparse heterosexual networks we have generated in this project. However, there are some situations, where the desired network is not sparse, the algorithm can fail to exactly produce a network with the desired *BJD* matrix for the joint-degree distribution. When this happens, a second algorithm is used to rewire the network so that it will exactly match the desired bipartite joint-degree distribution for the number of partners that a person’s partners have.

#### Rewiring **SexNet**  for a given *BJD* matrix

**Fig. 2:**
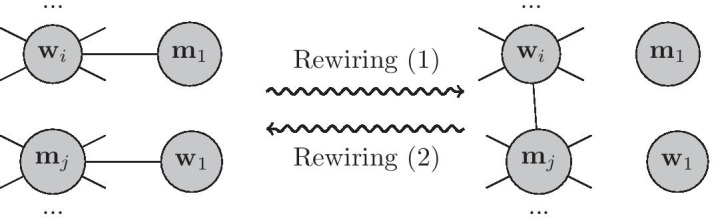
Schematic of steps 1 and 2 of Rewiring approach to correct *BJD*

The rewiring algorithm corrects any mismatch between the joint-degree distribution of generated **SexNet**  and the desired *BJD*. We define the joint-degree distribution of the generated **SexNet**  as ${\widetilde{BJD}}$ and the mismatch error matrix ${\mathcal {E}}=BJD-{\widetilde{BJD}}$. If the matrix ${\mathcal {E}}$ has nonzero elements, then the network is rewired to eliminate the error. There are three possible cases:

(i) If entry ${\mathcal {E}}_{(i,j)}, (i, j>1)$, is a positive value *k*, it means that **SexNet**  needs *k* more edges between degree *i* women and degree *j* men. To create these edges, we iterate the following process *k* times:
  - First, identify a woman, **w**$_i$, in **SexNet**, where $d_{\mathbf{SexNet}}({\mathbf{w}}_i)=i$, and has as a partner, **m**$_1$, with degree-1, i.e. $d_{\mathbf{SexNet}}({\mathbf{m}}_1)=1$.
  - Next, identify another man, **m**$_j \in {\mathbf{SocNet}} .N({\mathbf{w}}_i)-\mathbf{SexNet} .N({\mathbf{w}}_i)$, where $d_{\mathbf{SexNet}}({\mathbf{m}}_j)=j$ and **m**$_j$ has a degree-1 partner, **w**$_1$. That is $d_{\mathbf{SexNet}}({\mathbf{w}}_1)=1$.
  - Finally, we rewire the network by removing the edges (**w**$_i$,**m**$_1$) and (**m**$_j$,**w**$_i$) and add edge (**w**$_i$, **m**$_j$), as illustrated in the Rewiring (1) of the Fig. 2.
(ii) If element ${\mathcal {E}}_{(i,j)}$, $(i, j>1)$, is a negative value $k^{\prime }$, it means that **SexNet**  have extra $k^{\prime }$ edges between degree *i* women and degree *j* men. To remove these edges, we iterate following process $k^{\prime }$ times:
  - First, identify a woman, **w**$_i$, in **SexNet**, where $d_{\mathbf{SexNet}}({\mathbf{w}}_i)=i$, which has a degree-j partner like **m**$_j$, that is $d_{\mathbf{SexNet}}({\mathbf{m}}_j)=j$. The nodes **w**_*i*_ and **m**_*j*_ are selected so that they have at least one social contact with opposite sex that is not their sexual partner.
  - Next, identify another man, **m**$_1\in {\mathbf{SocNet}} .N({\mathbf{w}}_i)-{\mathbf{SexNet}} .N({\mathbf{w}}_i)$, and a woman, **w**$_1\in {\mathbf{SocNet}} .N({\mathbf{m}}_j)-\mathbf{SexNet} .N({\mathbf{m}}_j)$.
  - Finally, rewire the network by removing the edge (**w**_*i*_,**m**_*j*_) and add edges (**w**_*i*_, **m**_1_) and (**w**_1_,**m**_*j*_), as illustrated in the Rewiring (2) of the Fig. 2.
(iii) In the previous steps, we pushed back nonzero elements in ${\mathcal {E}}$ to its first row and column, which causes new nonzero elements in the first row and column. To remove these nonzero values, we have to add or remove small components. For example, if the element (i,1) of ${\mathcal {E}}$ is a positive value *k*, it means that we need a small component of a degree *i* woman whose partners are all degree 1 men. Therefore, we simply make this component from the people who are not currently in **SexNet**.*n*. If the element (1, *j*) of ${\mathcal {E}}$ is a negative value *k*, it means we have to remove a small component of a degree *j* man whose partners are all degree 1 women. Therefore, we simply look for such a component and remove it from **SexNet**.

We have found that this algorithm almost always converges to the desired *BJD*. However, there are rare cases when the desired rewiring nodes may not exist, and algorithm stalls with ${\mathcal {E}} \ne 0$. In test simulations, we could construct some sparse social networks where this algorithm could not exactly match the desired BJD. These cases were rare and, when they did occur, the rewiring step still improved the approximation to the desired BJD of **SexNet**. In the numerical simulations, all of the generated New Orleans heterosexual network had exactly the desired *BJD* based on survey data.

In the next section, we apply our algorithm to generate and analyze several random **SexNet**  corresponding to sexual activity of adolescent and young adult sexually active African Americans reside in New Orleans. First, we explain the inputs of our approach: New Orleans social network and joint-degree distribution- *BJD* matrix- corresponding to its sexual network. Then we use our algorithm to generate and analyze a bunch of **SexNet**s for a subpopulation of people in New Orleans.

## Simulations

We analyze an ensemble of sexual networks with a prescribed joint-degree distribution representing sexual activity of young adult African Americans in New Orleans.

### The New Orleans social activity data and **SocNet**

The **SocNet**  is based on the synthetic data generated by Simfrastructure (Eubank et al. 2010; Eubank 2008) for 130,000 synthetic people residing in New Orleans. Simfrastructure is a high-performance, service-oriented, agent-based simulation system, representing and analyzing interdependent infrastructures. The data for the network includes information for each individual, identified by their **PID** (personal identifier), and includes their age, gender, household, and other demographic information. The contact information for each PID is encoded in the contact file in Table 3.

**Table 3:** Table input for the social contact network of individuals

PID	FID	A	T
43722	16981	H	0.3
	11462	W	0.2
	37790	Sh	0.01
	$\vdots$	$\vdots$	$\vdots$
51981	23476	Sc	0.16
	18462	O	0.02
	10790	H	0.5
	$\vdots$	$\vdots$	$\vdots$
$\vdots$	$\vdots$	$\vdots$	$\vdots$

The PID is personal ID, FID is their social contact ID, A is activity in which PID meet FID, and T is the fraction of time in a day that two social contacts meet with each other through activity A. We have five different activities, including H as home, W as work, Sc as school, Sh as shopping, and O as others. For example, person 43722 stays with person 16981 at the same home for 7.2 h in a day

The Simfrastructure data was used to generate the original **SocNet**, which was then used to generate **ESocNet**  and **BSocNet**  as described in the previous section.

### The *BJD* matrix for New Orleans heterosexual activity

An ongoing community-based pilot study was conducted among sexually active African Americans ages $15-25$ in New Orleans (Kissinger 2014), to assess the effectiveness of prevention and intervention programs for chlamydia. Socio-demographic information including age, race, educational level and, sexual behavior—number and age of heterosexual partners in the past two months—and history of their STI test results were collected from 202 men and 414 women participants. Meanwhile, their partners’ information has been collected by asking questions referring to the status of each relationship such as the partner’s age and the possibility that their partner(s) have intercourse with others. The survey results were used to construct the *BJD* matrix of a heterosexual network of individuals in New Orleans. For a population $P = 15{,}000$ sexually active young adult men and their women partners residing in New Orleans, we have

$$\begin{aligned} {BJD_{15000} = \left( {\begin{array}{*{21}c} 1663 &\quad 1588 &\quad 1225 &\quad 896 &\quad 645 &\quad 469&\quad 342 &\quad 252 &\quad 186 &\quad 138 &\quad 105 &\quad 90&\quad 72&\quad 57&\quad 41 &\quad 34 &\quad 23 &\quad 24&\quad 14&\quad 13&\quad 14\\ 474 &\quad 452 &\quad 350 &\quad 255 &\quad 185 &\quad 133&\quad 97 &\quad 73 &\quad 52 &\quad 39 &\quad 31 &\quad 26&\quad 20&\quad 17&\quad 11&\quad 9&\quad 8&\quad 7&\quad 3&\quad 4&\quad 4\\ 198 &\quad 188 &\quad 145 &\quad 107 &\quad 77 &\quad 57 &\quad 40 &\quad 29 &\quad 22 &\quad 16 &\quad 12 &\quad 12&\quad 9&\quad 6&\quad 5&\quad 3&\quad 2&\quad 4&\quad 1&\quad 1&\quad 2\\ 68 &\quad 63 &\quad 49 &\quad 35 &\quad 26 &\quad 18&\quad 15 &\quad 10 &\quad 8 &\quad 6 &\quad 4 &\quad 3&\quad 3&\quad 3&\quad 1&\quad 2&\quad 0&\quad 0&\quad 1&\quad 10&\quad 0\\ 18 &\quad 18 &\quad 13 &\quad 9 &\quad 6 &\quad 6 &\quad 3 &\quad 4 &\quad 1 &\quad 1 &\quad 2 &\quad 0&\quad 0&\quad 1&\quad 1&\quad 0&\quad 1&\quad 0&\quad 0&\quad 1&\quad 0\\ 3 &\quad 3 &\quad 3 &\quad 2 &\quad 1 &\quad 1 &\quad 0 &\quad 0 &\quad 1 &\quad 0 &\quad 0 &\quad 1&\quad 0&\quad 0&\quad 1&\quad 0&\quad 0&\quad 1&\quad 0&\quad 0&\quad 1\\ \end{array} }\right) . } \end{aligned}$$

The dimension of this *BJD* matrix is $6\times 21$, that is, the maximum number of partners women have is 6 and for men is 21.

### **SexNet**  analysis

Using the social network and *BJD* matrix provided in the previous subsections and approach described in Section “Materials and method”, we generated 150 **SexNet**s of 15000 people for $p=0.2,0.4,0.6,0.8$ and 1, where *p* is the proportion of sexual partners that are selected from social friends. That is, 30 of the **SexNet**s are 20%, 30 are 40%, 30 are 60%, 30 are 80%, and the rest 30 are 100% subgraph of **BSocNet**. We then compared some descriptive metrics of this ensemble of random networks that were not imposed when generating the networks, including the size of giant components and bi-components, number of connected components, and average redundancy coefficient.

**Fig. 3:**
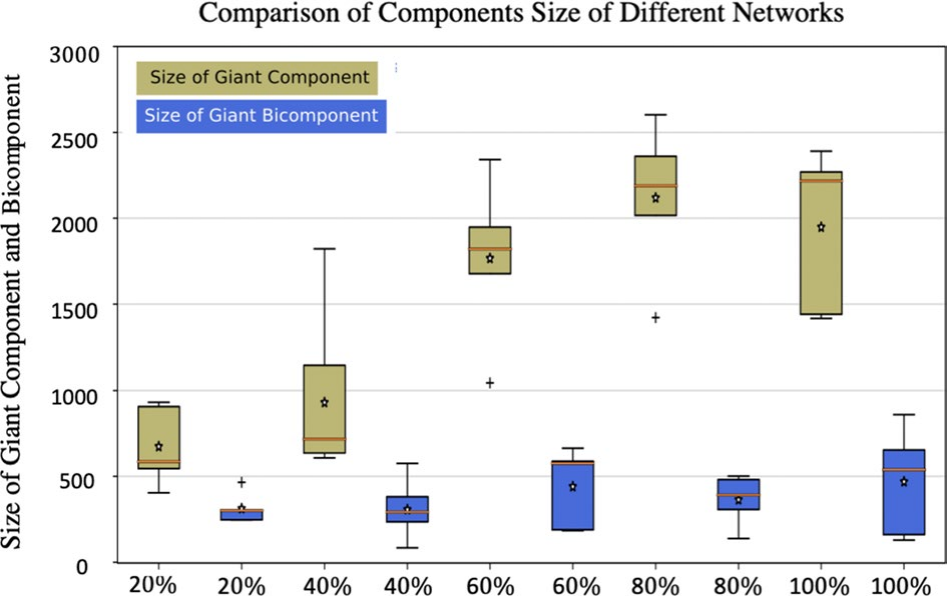
Box plot representing the size of the giant component and bi-component for each group of networks: x-axis are the level of the subgraph of **SocNet**. For example the value $20\%$ refers to the **SexNet**s which 20% of their edges comes from **SocNet**. The stars are the average values for each box and the orange line are their median values. The size of the giant component becomes bigger when the portion of the subgraph becomes stronger, however, the social network does not have much impact on the size of the giant bi-component

First, we evaluated and compared the size of the giant component and bi-component (the first and second biggest connected components of the network) for each group of the networks. Figure 3 shows the box plot of these sizes: there is an increment in the size of giant components when people select most of their sexual partners from their social contacts. Because in that case, sexually active people are tighter together within the social contact network. But there is not a significant difference in the size of giant bi-component.

**Fig. 4:**
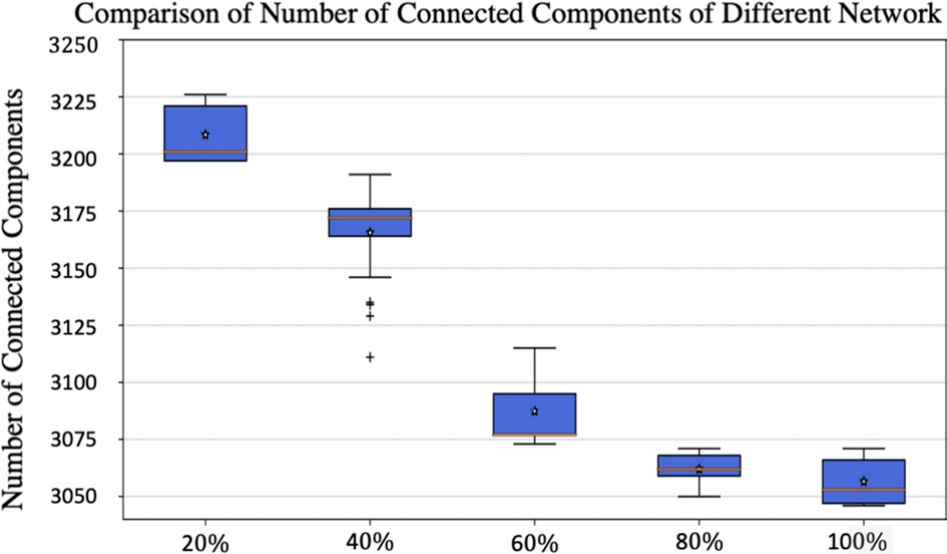
Box plot representing the number of connected components, *Nc*, for each group of networks: x-axis are the level of the subgraph of **SocNet**, for example the value $20\%$ refers to the **SexNet**s which $20\%$ of their edges comes from **SocNet**. The stars are the average values for each box and the orange line are their median values. A significant difference is observed in *Nc* between each group, *Nc* is lower in larger subgraph of social networks

The number of connected components, *Nc*, is another measure characterizing network toughness. This measure can be, not necessarily, correlated to the component’s size of the network. Figure 4 displays descriptive statistics for *Nc* in each network group. Note that data distributions are approximately symmetrical, and metrics of *Nc* are similar across groups, but, they change by changing the source of partner selection- changing *p*.

Redundancy coefficients are the measure of the degree to which nodes in a bipartite graph tend to cluster together:

#### Definition 1

For a bipartite network, redundancy for a node is the ratio of its overlap to its maximum possible overlap according to its degree. The overlap of a node is the number of pairs of neighbors that have mutual neighbors themselves, other than that node (Latapy et al. 2008). For a typical node **v**, the redundancy coefficient of **v** is defined as

$$\begin{aligned} Rc({\mathbf{v}})=\frac{|{\{}{\{} {\mathbf{u}}, w {\}} \subseteq N({\mathbf{v}}), \exists {\mathbf{v}}^{\prime }\ne {\mathbf{v}} s.t \mathbf{uv }^{\prime }\in {\mathbf{E}}, {\mathbf{wv}}^{\prime }\in {\mathbf{E}} {\}}|}{{\frac{|N({\mathbf{v}})|(|N({\mathbf{v}})|-1)}{2}}}, \end{aligned}$$

where, $N({\mathbf{v}})$ is the set of all neighbors of node **v**, and **E** is the set of all edges in the network.

**Fig. 5:**
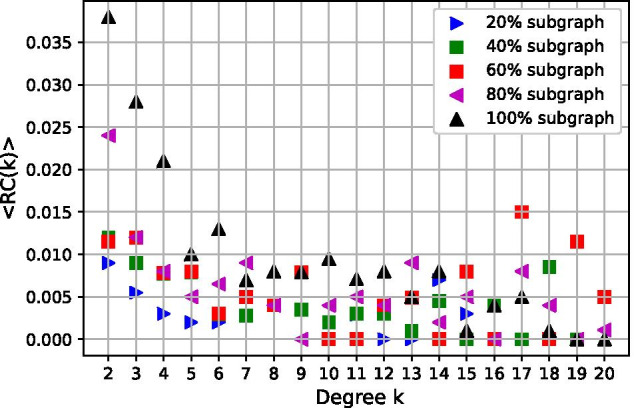
Scatter plot of Redundancy Coefficient *Rc* versus degree for five different networks: *Rc* for **SexNet**  which is strong subgraph of **BSocNet**  (higher percentage of subgraph *p*) is higher, because clustering coefficient for **BSocNet**  is high and therefore, **SexNet**  inherits this property by having higher *Rc* than the ones which are weak subgraph of **BSocNet**

We compare this measure for the networks in Fig. 5: each data point *Rc*(*k*) for degree *k* is obtained by averaging redundancy coefficient over the group of people with *k* partners. In most of the networks, *Rc*(*k*) decreases with *k* (Newman 2010). Redundancy coefficient *Rc* is affected by social network **BSocNet**: when people select more sexual partners from their social contacts the value for *Rc* increases, which is because of stage one of the algorithm- Generate an extended social network. In that stage, by connecting the social contacts of a person in **BSocNet**, we increase its clustering coefficient. Therefore, because increasing *p* **SexNet**  becomes a stronger subgraph of **BSocNet**, it inherits more properties from **BSocNet**, that is, by increasing *p* *Rc* of the **SexNet**, which is correlated to clustering coefficient of **BSocNet**, increases.

## Discussion

We described a new algorithm to generate an ensemble of heterosexual networks based on heterosexual behavior surveys for the young adult African American population in New Orleans. The prescribed degree and joint-degree distribution represented the heterosexual network embedded within a social network that captures the biased mixing of the population based on age, physical location, and social activities.

We generated an ensemble of different heterosexual networks with the same *BJD*. When the networks had a more percentage of partners selected from their extended social contacts, then we observed a tighter distribution in the number of connected components and the size of giant component and bi-components. When more partners are chosen from the extended social network, instead of randomly selected from the population, then the size of giant component increases, and following that the number of connected components decreases, which is because of reducing the mixing in generating sexual network: when people select their sexual partners from their social contacts they stand in a tight group within social network. In fact, when being subgraph of the extended social network become stronger, the candidate set of sexual partners for each person that is set of social contacts decreases and becomes local (this set includes close contacts and contacts of contacts) compared with when this set is the whole population.

As *p* increases, then more partners are chosen from a person’s extended social network. This also increases the network clustering coefficients (where more partners of your partner’s partner are also one of your partners). The redundancy coefficients for networks increases as the dependence of sexual network on social one rises when *p* increases, which is because of the high clustering coefficient of the social network due to the first stage of the algorithm, generate an extended social contact network. In that stage, we made some new contacts between the contacts of each individual, which causes the increment in the clustering coefficient of the social contact network. On the other hand, when more partners are chosen from the social contact network, more properties of the social network such as the clustering coefficient become inherited by **SexNet**. Thus, increasing *p*, we observe increment in the redundancy coefficient of **SexNet**.

We studied the metrics of networks that may affect the spread of an STI at the population level. Metrics such as the size of giant components and redundancy coefficients provide information about connectivity among the individuals. In our future work, when studying the spread of chlamydia on heterosexual networks, we will measure their impact on the prevalence of chlamydia over **SexNet**s generated using different *p* values.

Our bipartite networks are being used to study the spread of chlamydia in situations where the probability of infection spread via homosexual activities is negligible. The approach can be extended to model the spread of STIs with mixed heterosexual, bisexual, and homosexual activities such as the spread HIV/AIDS. This can be accomplished by modifying the second stage of the algorithm to include the fractions of the population within these different sexual groupings.

There are still unanswered questions for proving the existence of a heterosexual network with a prescribed joint-degree distribution embedded within a prescribed social network. That is, there are no explicit criteria to guarantee that a heterosexual network with a particular joint-degree distribution can be embedded within a particular social network or not.

We are currently simulating a stochastic agent-based network model on **SexNet**  for the spread of chlamydia and comparing different intervention strategies to control the spread of STIs that are implemented in public health, such as screening, partner treatment, rescreening, and peer referrals (Qu et al. 2020; Azizi et al. 2020). These simulations will use the underlying social contact network to improve the current intervention models by considering the impact of counseling and behavioral changes such as increasing condom use or social contact notification.

## Data Availability

The datasets used during the current study are available from the corresponding author on reasonable request.

## Abbreviations

STI: Sexually transmitted infection
**SocNet**: Social contact network
*BJD*: Bipartite joint degree
**ESocNet**: Extended social contact network
**BSocNet**: Social bipartite network
**SexNet**: Sexual network
**PID**: Personal identifier
**FID**: Friend (social contact) identifier
*Nc*: Number of connected component
*Rc*: Redundancy coefficient
